# Multidisciplinary Firms and the Treatment of Chronic Pain: A Case Study of Low Back Pain

**DOI:** 10.3389/fpain.2021.781433

**Published:** 2021-11-10

**Authors:** Julie G. Pilitsis, Olga Khazen, Nikolai G. Wenzel

**Affiliations:** ^1^Department of Neurosurgery, Albany Medical College, Albany, NY, United States; ^2^Department of Neuroscience and Experimental Therapeutics, Albany, NY, United States; ^3^Broadwell College of Business and Economics, Fayetteville State University, Fayetteville, NC, United States

**Keywords:** multidisciplinary pain clinic, cost sharing, chronic pain market, chronic pain, acute pain

## Abstract

Sixteen million people suffer with chronic low back pain and related healthcare expenditures can be as high as $USD 635 billion. Current pain treatments help a significant number of acute pain patients, allowing them to obtain various treatments and then “exit the market for pain services” quickly. However, chronic patients remain in pain and need multiple, varying treatments over time. Often, a single pain provider does not oversee their care. Here, we analyze the current pain market and suggest ways to establish a new treatment paradigm. We posit that more cost effective treatment and better pain relief can be achieved with multi-disciplinary care with a provider team overseeing care.

## Introduction

The cost of chronic pain to our society is staggering and has been quoted as up to $USD 635 billion in health care expenditures, disability and loss of productivity (1). Pain diagnoses make up 4 of the top 10 reasons people seek medical care (2). Costs for health care utilization include hospital admissions, emergency department visits, and expensive invasive therapies. Societal costs include missed workdays and impact on family dynamics, which may also have economic ramifications. Chronic pain affects roughly 20 million adults in the United States and has a profound influence on an individual's productivity, quality of life and mental health (3–7). Sixteen million suffer with chronic low back pain.

Chronic pain is defined as pain that lasts more than 3 months despite treatment. Patients with chronic pain are desperate to find a solution for managing their pain, but only half of these patients report having control over their pain (8). The issues surrounding management of chronic pain are complicated. What works for acute pain often does not work for chronic pain. For the purpose of this article, we will focus on the market for low back pain relief, which exceeds $100 billion USD (9, 10). We will begin by defining the patient population constituting the pain relief market and addressing the current state of the consumers and service providers in the market. We will then examine multidisciplinary clinics as a potential solution and address mechanisms of establishing financial sustainability and limiting barriers. Section Introduction describes the current market for pain relief. Section Background presents an alternative model, multidisciplinary firms for comprehensive chronic pain management.

## Background

### The Consumers in the Market of Pain Relief

Patients with acute low back pain are often desperate to obtain pain relief and will often seek any number of medications or devices to achieve that relief (i.e., over the counter pills or patches, assistive devices and braces and even more expensive alternatives like tilt tables). They may then go for a massage, to a chiropractor, or to an acupuncturist. In the vast majority of patients, the acute pain dissipates within 2 weeks (11) and they are no longer consumers in the market for pain relief. Those who are unaware of this natural history of the disease, are in extreme pain, and/or are still in pain after 2 weeks, may go to the emergency department or see their primary care doctor who will likely prescribe physical therapy and medications (12).

Up to 80% of acute pain exacerbations improve within 6 weeks (13), at which point the consumers leave the market for pain relief. For the subset who have continued pain, they may be referred to a pain specialist who generally focuses on injections (14). Alternatively or in addition, an MRI may be ordered after a course of physical therapy (15). The MRI is very rarely read as normal, and though the number of findings on lumbar MRIs in people with pain and without pain are similar (16), review of an imaging report often leads to a trip to the neurosurgeon or orthopedic spine surgeon. In most conservative spine practices, the majority of patients are not surgical candidates and are referred for injections or other conservative measures.

By 12 weeks, 95% of patients have resolution of pain (13). Over the course of a lifetime, a person may have acute pain that requires entry into the pain market several times with symptoms of varying intensities (i.e., a trip to a pharmacy for a heating pad/ibuprofen for acute back spasms v. a course of chiropractic manipulation for pain that persists for several weeks v. a spine surgery for acute leg pain associated with a herniated disc). However, in most cases, the pain is acute. The remainder of consumers, that is those who have no relief, are termed chronic pain patients and remain in the market for chronic pain relief services.

### The Current State of the Market for Pain Relief in Chronic Pain Patients

Chronic back pain patients become very familiar with multi-modality treatment that consists of medications, physical therapy, alternative therapies, injections, surgery, and/or neuromodulation. Though some medications for the treatment of chronic pain are reasonably priced, they often have notable side effects and are not well tolerated by patients. The relative low cost of opioids, in part, contributes to their overuse (17) despite significant evidence that these drugs are not appropriate for the majority of chronic pain sufferers. Other types of medications (e.g., pregabalin, lidocaine patches) can be quite expensive, ranging from $USD 250–1,000 per month for treatment.

Often, physical therapy and core-strengthening/stretching may actually be more effective than medications (18, 19). However, patients are often more eager to try a medication, than an alternative therapy, as the price of medications is often covered in large by insurance, while alternatives are typically not. Further, there are psychological barriers to such behaviors-people who have difficulty moving fear they will be asked to do just that at therapy. Additionally, it takes a 6–12 week commitment to daily exercises to see a difference. Finally, patients are typically responsible for co-payments associated with physical therapy which can be cost prohibitive (i.e., $40 per visit with visits 3x/week for up to 20 visits).

Other forms of physical manipulation have even greater financial barriers as acupuncture and chiropractic are often not covered by insurance. Biofeedback and cognitive behavioral therapy, though proven beneficial for chronic pain, are often met with resistance by patients who feel like clinicians are telling them their pain is not real. In addition, there are significant barriers for pain patients obtaining psychological evaluation and follow-up due to a relative paucity of psychologists who see patients with chronic pain and psychologists/psychiatrists in general (20).

Ultimately, for the reasons above and human nature, patients often look for a quick fix which may or may not be beneficial depending on the pathology and the procedure chosen. Patients will often see pain management providers who more commonly offer invasive options instead of medications or body therapies, based on their area of expertise (21). The co-payment for one visit for steroid injections or radiofrequency ablations is similar to that of one visit at physical therapy. As these injections and ablations are only performed 3–4x a year and have more immediate results, patients have financial benefits for undergoing these therapies. When injections are no longer helpful, surgery is often considered.

If there is a primary issue with the spine, an appropriate surgery may be performed. There is variability in what type of surgery, if any, will be offered from surgeon to surgeon and region to region. Patients often have a co-pay anywhere between $50–1,000. The cost submitted to insurance for the procedure however can range from $USD 10,000 to over 100,000 depending on the type of surgery performed (22). Despite the above attempts, it is fairly rare that patients with chronic pain have complete resolution of their pain.

After a year or two of repeating the cycle above, patients may then move onto more neuromodulation therapies- that is, treatments that alter their perception of the sensation. These therapies may include spinal cord stimulation or intrathecal pumps. These procedures are successful in 50-80% of people, where success is defined as 50% pain relief (23). However, failures may occur in up to 30% of patients and 10–15% of the devices are explanted (24, 25). These devices cost an average of $30,000 per implant and the total cost of surgery/hospitalization is often ~$50,000. Neuromodulation therapy has experienced a growth rate of 20% a year, which is outside the growth rate for other treatments. Thus, insurance companies are beginning to become more stringent in defining which patients will be covered. Additionally, neuromodulation has been shown to be more cost-effective compared to conservative treatments (4, 26–28) and spine reoperation in properly selected patients (29). In these cases, health care resource utilization decreases (4, 6, 26, 30, 31), indicating successful exit from the pain market. However, in patients that ultimately do not have success with the therapy and have the device explanted, total costs are higher (32). Newer technologies are also available. Currently, these include stem cell therapies injected into the spine (33) or minimally invasive procedures performed by pain physicians (34–37). Evidence remains limited at this point.

### Flaws in the Current Market for Chronic Pain Relief

Often, regardless of what therapies the chronic back pain patient has undergone, there is continued healthcare utilization, with the majority of spine and spinal cord stimulation patients requiring multiple medications and/or cycles of injections, physical therapy, and/or surgery over time (25, 38). Based on this pattern, the patients that remain in debilitating pain, and the healthcare expenditures associated with this suboptimal care (39), there is little debate that the market for chronic pain relief is flawed. To analyze the market, we will examine it in economic terms.

The consumer is the patient in pain. The market generally works well for the acute pain patient, as 95% of these patients exit the market by 12 weeks (13). However, for the 5% who remain in the market, it works less well, and in some cases, leads to debilitating pain that precludes normal activity for decades. The service providers in the market (which will be referred to as “firms” for the remainder of this paper) generally specialize in one type of therapy. This categorization is a slight simplification as there are nuances among different therapies – but they generally fall into the same category of therapies. For example, body work, physical therapy, chiropractic, acupuncture, and/or massage are generally provided by specialty vendors who provide one service. Pain specialists, as a trend in the last 5 years, routinely perform injections and often only provide medications in special cases (8, 38). Pain psychologists are few and far between and waiting lists are long (20, 40). Surgeons, of course, specialize in surgery. For all firms, the inputs are chronic pain patients and the ideal outputs are chronic pain patients with manageable levels of pain. It is important to note that once patients are in chronic pain, they often will have some degree of pain even when treatment is optimized (41).

Unfortunately, the output is often not achieved within the current market. More commonly, patients have some degree of pain relief with one therapy and move onto treatment by the next firm, leaving patients to continue in the pain relief market with no single physician specializing in pain management overseeing their care plan. Further, the market is limited by resources. As treatment of chronic pain is difficult and may lead to physician burnout (42), there are limited numbers of providers willing to take care of these patients. Often, care remains with the primary care physician, who often does not have the time and/or resources to manage these patients.

In the last decade, another barrier has arisen. In some cases, the primary care physician and the patient have found a medication regimen that works “well enough.” A subset of these patients have been treated successfully with low dose opioids for a decade. This option, though not recommended for the vast majority of patients, is highly successful in a small minority (43, 44). Recent regulations and stigma related to opioids preclude many providers from continuing medications. Patients are subsequently left without therapy and they re-enter the pain market where they may see a pain specialist. Most physicians consider opioid prescribing a highly undesirable risk-to-benefit ratio (not only for patients but also for them), hence, most primary care and pain practices have become very limited in their ability and desire to prescribe (45). Prescribing responsibly requires pain contracts, urine drug screens, checking databases for inappropriate use, and frequent appointments to receive a limited supply of medications (8). Thus, there is a significant transaction cost to firms which prescribe. As the cost of caring for patients on opioids is ~70% higher than for those not on opioids, it is not fiscally wise to continue this practice (46). The added risk of staff burnout compounds the issue (42).

What has resulted is patients having more costly procedures that may be less effective. Patients become frustrated, feel deserted, and look for practices which prescribe medications. There are many practices that focus on prescribing opioids in a reasonable fashion and have either found ways to make this financially viable or are part of a tertiary care facility or state facility which focuses on serving public health needs. However, there are also practices that have been described as “pill mills.” In these businesses, the profit associated with writing prescriptions/drug screens may complicate motivation to wean patients to the lowest dose or off medications entirely, even if they have pain relief (47). Fortunately, these practices have been investigated by law enforcement in the last decade. To be fair, these are not the only pain practices where ethics become complicated. Most firms in the pain market rely on volume due to low revenue margins (48). Incentivizing physicians or any health care provider to see more patients is similar to examples of factory line workers being incentivized to make more parts (49). Quality dissipates with this type of incentive (50).

Taken together, while the current market may work for the acute pain patient with a straightforward problem, it does not fulfill the needs of the chronic pain patient. We posit that concentrating care into comprehensive, multidisciplinary firms would be highly beneficial and would likely lead to improved patient outcomes and reduced healthcare expenditures.

## A Proposed Model: Multidisciplinary Firms

We have already seen the overall costs of chronic pain. For the 16 million Americans who have chronic back pain, expenses are estimated at $USD100 billion (9, 10). This staggering figure includes both health care expenditures and estimated lost time and wages. Specifically, patients with chronic pain who are not adequately treated are likely to have more disability over time and subsequently will have an increase in healthcare resource utilization (HCRU) compared to patients who have been adequately treated (3, 4, 51). Emergency room (ER) visits can be a surrogate marker of HCRU and it has been found that 42% of visits are due to pain (51, 52). Further, chronic pain patients with more disability use the ER more than those who have greater function (51, 53, 54). They may visit as often as twice to three times a month due to uncontrolled pain and/or lack of an established care team (52). Overall, multidisciplinary pain clinics have been shown to reduce ER visits (55, 56).

Outside of the ER, multidisciplinary pain clinics have resulted in cost savings of $6.68 per day in prescription costs (57). The implementation of a multidisciplinary pain clinic at Geisinger (58) resulted in decreases in the number of primary care visits, acute inpatient admission rates, opioid prescription fill frequencies, and the use of high-end diagnostic imaging, which corresponded with a reduction in total medical costs (59). Such collaborative clinics have been found to positively alter patients' care-seeking behaviors (57, 59).

Most importantly, patients in multidisciplinary clinics have better outcomes. These patients generally report less pain, have fewer effects of pain on activity and have more appropriate use of non-opioid medications (60, 61). They have regularly scheduled outpatient follow-up, (60–62) greater health literacy about pain, and reasonable expectations for relief. However, pain management is often viewed as low priority due to the stigma that pain is a symptom not a disease (63). Compounded with fears of increased costs for payers and providers (57), multidisciplinary pain clinics are not prioritized. Currently there is little to no incentive for both payers and providers to be involved in the implementation of these clinics, despite the benefits. However, in order for a multidisciplinary clinic to be successful, thoughtful deliberation between all clinicians involved is necessary to develop a protocol/ pathway for pain patients. We have done this for our pelvic pain patients (64). Similar could be done with different types of pain leading to low back pain, including SI joint dysfunction, mechanical pain, and neuropathic pain.

### Opportunity Costs and Financial Sustainability

When thinking about developing a multidisciplinary pain clinic, it is necessary for all those involved in treatment of chronic pain to reach a consensus on the organizational structure of the clinic. This requires an assessment of internal strengths and weaknesses and external threats and opportunities. Much depends on what already exists in the community and where the needs are. A bit depends on the demographics of the community and appropriately determining who would be a candidate. In a community with limited resources and a large demographic of manual laborers who have performed decades of heavy lifting, there is a mismatch between resources and need. A patient who has undergone three courses of physical therapy, three courses of injections, four spine surgeries and 10 MRIs, would be appropriately served in this clinic. The patient who has only had one of the four interventions, no matter how many times, may or may not be a candidate. In a community with more resources, a patient after one surgery with continued pain may be appropriate. Each community should work together to perform a needs assessment.

This point would warrant discussion and depend on the other firms in the market, the capacity of the multidisciplinary clinic and the number of chronic pain patients the market serves. However, in the majority of markets, it is likely that the input to the firm (e.g., a patient with one spine surgery) would still be too high, based on the firm's resources for achieving the desired output. To avoid overwhelming the system, a more reasonable entry point into the pain market may be after 6 months of pain and the failure of two therapies. Additional data is needed to test this hypothesis and again may vary from community to community. The multidisciplinary firm we discuss in this article focuses on back pain, but it is important to note that the entry point should be altered depending on the disease process and the external market. For example, all patients with pelvic pain that come into care with any of our providers can be entered into the system because there are so few resources available to these patients that they often have been suffering for more than a decade before they seek care (64). Patients with cancer pain also would need to enter into the system much more quickly (65). Each region should work together to perform a needs assessment.

### Partnerships With Insurance Companies and Other Firms in the Market

Third-party payers are essential stakeholders in the discussion. The most established multidisciplinary program for chronic pain has been at the (66, 67). More recently, regions such as Eastern Pennsylvania have insurance companies that have partnered with health systems (2019). In 2012 Piedmont and WellStar healthcare systems created a joint venture called the Georgia Health Collaborative which utilizes a care model with the goal of implementing both prevention and care management programs with higher quality care at lower costs (68).

Partnerships with insurance companies in these initiatives has increased (62). Such practices have also helped in monitoring financial risk to manage healthcare spending and potential losses, while also providing patients with better, more well-rounded care (69). In 2014, Geisinger (58) implemented a multidisciplinary pain clinic, noting significant reduction in health care utilization and cost of care (59). Recent partnerships between Southeast MI hospitals and Blue Cross Blue Shield of MI, have resulted in more implementation of multidisciplinary teams, beginning with integrating clinical pharmacists into the patient-care team (70). These teams have led to improved resource utilization.

However, in most regions, partnerships for development of multidisciplinary clinics for pain have not been pursued presumably due to cost and volume concerns (57). To determine what is and what is not possible requires discussions among the groups. A discussion would first lead to a better-defined partnership between the insurers and the hospitals. Expectations must be established and the poor results that are achieved with the existing market understood, further, all must accept that *adequate* pain relief, rather than *total* pain relief is the goal (71). Next, the group could determine the need and timing for certain therapies and limit the fairly common “fail first” model prior to granting authorization for some diagnostics and therapies when the model does not promote better care (72). Clinicians, researchers and economists must show the cost-benefit analysis of these programs for payers to desire them (73–75). Additional payment structure that is mutually agreed upon must be established so that groups providing the care to patients can cover costs and an agreed-upon margin (76).

Additionally, some degree of intervention from insurance companies and large firms will be needed to encourage providers to use the multidisciplinary clinic. Within geographic regions, how this will be done and which patients will be included will vary. Limiting pre-authorizations or encouragement through pay-for-performance may be some means of promoting the use of multidisciplinary clinics. Ultimately, for this model to be sustainable, the multidisciplinary firm will have to develop a comparative advantage and provide the best outputs. As only 27% of patients with chronic pain have Medicare, the process is likely to start with commercial payers (77).

Of course, in addition to partnership with insurance companies that mandate their patients participate, the multidisciplinary clinic needs additional patient inputs. Including firms in the process of caring for patients will likely add to this input. Once a care plan is developed by the multidisciplinary clinic as described above, it could be subsequently implemented in combination with other firms to ensure inputs, internal capacity, and financial viability for the surrounding market. This set-up would allow patients to be treated closer to their home when all other factors are equivalent. Alternatively, for niche services, the patients may need to receive the services within the multidisciplinary group. Careful cost analysis must be done to ensure that capacity is appropriate and that the use of internal and external services is a financially sustainable model. Oversight will need to be maintained by the multidisciplinary clinic on patient outcomes. This could be done through semi-annual or annual assessment.

Development of multidisciplinary teams may be hindered by physicians themselves. Sometimes physicians are reluctant to give up patients to a multi-disciplinary center and/or do not have the administrative a number of providers who have very busy practices and necessitates personnel for coordination (78). Further, some practices may be in direct competition. Additionally, there is limited interoperability between medical records in different practices and systems (79, 80). How records will be housed and shared will need to be determined and subject to regulations.

### Minimizing Transaction Costs

When establishing a new type of practice, ease of the referral process is essential (81). Sometimes specialized firms within a tertiary medical center make referrals difficult. In this case, the external firms, which are much more available, maintain considerable market share. Patients face similar challenges obtaining appointments. Put in economic terms, the transaction costs of dealing with a tertiary medical center can be too great for many referring providers and for many patients. The buildings are huge, the waits are long, the providers seem overworked, and the telephone trees are unwieldly. A multidisciplinary clinic may also not serve the needs of the primary care doctor if only recommendations are made and the primary responsibility for patient care still lies with the front line physician. Primary care physicians are often overworked and undercompensated and cannot afford to take care of chronic pain patients, or find it unduly difficult. If pain practices take this burden from them, the output is less important and the referrals grow.

In establishing our multidisciplinary pelvic pain health consortium at our institution-Albany Medical Center, we have found that providing a single contact with an email and phone number works well for providers (64). Intake is then performed by that one patient navigator, using a form that was developed by the group as a whole and appropriate consultations are embarked upon. We have been able to initiate these plans over the phone, virtually, or in person. Then, in a mature multidisciplinary group, the team should describe what the expected outcomes are for the patient, their family and their primary care physician. The multidisciplinary group should discuss all patients that have entered the clinic and who are not achieving the expected outcomes at weekly and/or monthly conferences. Often, these discussions also help providers stay on message in discussion with patients. For example, patients with lower health literacy may be highly resistant to working with pain psychologists as they feel that referral is a sign that their providers think “the pain is in their head.” They fail to realize that pain is a tridimensional (bio-psycho social) experience involving sensory, cognitive and emotional components (82) and further do not realize that biofeedback, talking about coping and strategies that empower them to independently control their pain may be valuable (8). Finally, as pain changes over time and new advances are commonplace, multidisciplinary discussion may prompt new ideas, thus increasing the likelihood of success (38).

### The Importance of Cost-Sharing

An upfront investment is needed to ensure buy-in among all parties and to ensure that the multidisciplinary clinic's resources and capacity is adequate to provide the sources they are meant to provide. Creative means of gaining buy-in and cost-sharing will be needed. Partnerships between hospitals and insurance were discussed above. To hold the physician accountable, it is important to emphasize transparency and accountability of patient outcomes. Medicare Pay for Performance (P4P) strategies can be adapted for pain practices, based on metrics selected by pain providers, rather than metrics which are less relevant. This has been an issue for specialties in the early attempts of P4P. Quality in these metrics should initially be incentivized and, over time, penalized in order to make sure that the outputs are adequate.

Another option is patient cost sharing. which has been shown to reduce health care expenditures. However, it is important to note that the reduction does not differentiate between high- and low-value care (83). Further, in most areas of medicine, it is difficult to employ patient cost-sharing for all medical care in real life situations, because of the unpredictably of life. As these resources only cover the chronic pain the patient is suffering from and they do not affect the care patients would receive for other chronic conditions or for acute unexpected conditions, this system does not suffer from moral hazards that diffuse cost-sharing plans may suffer from Baicke et al. (83). Further, it is important to note that patients can become incentivized to suffer in pain (i.e., disability, workman's compensation, beneficial family dynamics). A counter-incentive to improve cost-sharing could better balance the patient's behavioral economics. Elasticities of demand vary by chronic conditions patients suffer from Chandra et al. (84) and where the elasticity of chronic pain services has not been defined. We know that dental and psychiatric services are more elastic than those of other medical services (83) for the general population. Interestingly, we would posit that chronic pain care would also be more elastic if there were not a motivation for a subset of patients to seek such services to continue receiving disability and workman's compensation benefits. It is important to note, however, that cost-sharing could be detrimental to patients in lower socio-economic brackets who have fewer monetary and non-monetary resources (84). A cost-sharing program could be on a sliding scale, depending on income relative to the poverty line [as has been done in Massachusetts (84)].

One potential strategy is to offer different co-payments depending on how likely a patient is to benefit from a therapy. If the patient is likely to benefit based on the medical literature, the co-pay would be lower. If the patient is unlikely to benefit or likely to have only a modest benefit, the co-pay would be higher. Unfortunately, in chronic pain the data is lacking as to which patients are likely to benefit and pain phenotypes are difficult to determine from the medical care as ICD10 codes do not accurately reflect patient status (85). Insurance companies restrict services when treatments are experimental. Insurance partnership with the multidisciplinary clinic may allow for physician input on low v. high probability of improvement for individual patients, especially where in chronic pain management a satisfactory outcome is considered 50% pain relief in 50% of patients (23).

Alternatively, patients could be given all the information and review it with their treatment providers and then make decisions in conjunction with their care team. They could be given a certain number of annual resources and determine how they will use those resources based on their pain. This strategy may be effective, as the patient and the multidisciplinary team are most likely to have the best insight into the patient phenotype. It would be essential for patients to be clear on the treatments and the cost sharing; thus, the plan must be straightforward and relatively simple (86). Additionally, cost-sharing for treatment would need to be done within the construct of a multidisciplinary clinic as care does not involve a single treatment. However, this may negate the draw to complementary services which have lower co-pays (83). We see this currently with patients opting for more invasive therapies. We also see this in providers when reimbursements for one procedure are far greater than for another. Taken together, cost-sharing could modulate behavioral economics while allowing for improved, more affordable care.

## Conclusion

Sixteen million suffer with chronic low back pain at an annual cost of $100 billion. The current market for pain relief does not meet the needs for patients for chronic low back pain. We posit that a multidisciplinary care clinic can be beneficial for the patients, providers, hospitals and insurance companies. Implementation will require appropriate partnerships and organizational structures. Opportunity costs, cost-sharing and relationship with external firms will vary regionally. Future work should examine best practices and expand this model from lower back pain to chronic pain, generally.

## Author Contributions

JP conceived of and coordinated the review and assisted in writing the manuscript. OK and NW also assisted in writing the manuscript. All authors contributed to manuscript editing, and approved the final submission.

## Conflict of Interest

JP is a consultant for Boston Scientific, Nevro, Medtronic, Saluda and Abbott and receives grant support from Medtronic, Boston Scientific, Abbott, Nevro, NIH 2R01CA166379-06 and NIH U44NS115111. She is the medical advisor for Aim Medical Robotics and Karuna and has stock equity. The remaining authors declare that the research was conducted in the absence of any commercial or financial relationships that could be construed as a potential conflict of interest.

## Publisher's Note

All claims expressed in this article are solely those of the authors and do not necessarily represent those of their affiliated organizations, or those of the publisher, the editors and the reviewers. Any product that may be evaluated in this article, or claim that may be made by its manufacturer, is not guaranteed or endorsed by the publisher.
